# Death of William Henry Morgan, D.D.S.

**Published:** 1901-06

**Authors:** 


### DEATH OF WILLIAM HENRY MORGAN, D.D.S.

INFORMATION of the death of Dr. Morgan came too late for extended notice in this number. This will appear in the July issue.

His departure, coming, as it has, almost immediately after the death of Dr. McKellops, is a silent but none the less positive reminder that the builders of our profession in the United States are fast passing away. Of these, Dr. Morgan was one of the most active and influential.

He died at his home in Nashville, Tenn., on Thursday morning, May 16, 1901.

